# Bridging policy and practice: exploring mental health assets in varsity athletes and non-athletes in Canada

**DOI:** 10.3389/fspor.2026.1800374

**Published:** 2026-08-19

**Authors:** Jessica Malcolm, Philip Sullivan

**Affiliations:** Centre for Healthy Youth Development through Sport, Brock University, St. Catharines, ON, Canada

**Keywords:** Canada, mental health assets, mental health literacy, post-secondary, resiliency, social support

## Abstract

Promoting mental health in elite athletic environments has become a priority. Recent research supports the promotion of mental health assets – individual-level resources that maximize the ability of individuals to attain and maintain mental health and wellbeing – as an underutilized approach to improving mental health in sport. The current study investigated the mental health assets of social support, resiliency, and mental health resource literacy among Canadian university students in relation to their gender and sports participation. Using data from the 2023 deployment of the Canadian Campus Wellbeing Survey (CCWS), we found that student-athletes (*n* = 1,143) reported significantly higher levels of all three mental health assets than non-athletes (*n* = 24,365); and that these differences were greater for men than women for resiliency and mental health literacy. These results add to the somewhat inconsistent literature regarding the mental health of intercollegiate athletes relative to their non-athlete peers. Furthermore, they support nuanced mental asset strategies for student-athletes.

## Introduction

Mental health among postsecondary students in Canada has emerged as an urgent priority and cause for concern (1, 2). Surveys have consistently shown high rates of stress, anxiety, and depression among university populations (3–5), with the 2019 Canadian sample of the National College Health Assessment (6) reporting that nearly 70% of students felt overwhelmed by anxiety and over half indicating a period of depression that interfered with daily functioning. These challenges are further compounded for student-athletes, who face unique pressures due to high-performance demands, hold dual academic and athletic roles, and may be reluctant to seek mental health support (2, 7, 8). As such, supporting the mental health of student-athletes in Canadian universities is both timely and critical.

Research on the mental health of student-athletes has produced somewhat inconsistent results. Davoren and Hwang (9) found that American student-athletes displayed lower levels of depression and anxiety than non-athletes, while Sullivan and colleagues (10) found that 20% of Canadian student-athletes showed scores of psychological distress indicative of severe mental illness. Similarly, while university athletes have been shown to display relatively high levels of mental health stigma and lower resource use (11), other studies have found that student-athletes display relatively higher service use and response to treatment (12). Overall, these inconsistencies may be best summarized by the conclusions of a systematic scoping review by Kegelaers and colleagues (13), who found that two-thirds of the studies in their review concluded that student-athlete mental health is equal to or worse than that of their non-athlete counterparts; while one-third found their mental health better.

U SPORTS is the governing body of intercollegiate sports in Canada. As per their regulations, student-athletes must complete three full courses during the academic year to maintain their eligibility. While scholarships are available, they are quite modest compared to other countries, particularly the NCAA in the United States. For example, in Ontario University Athletics (OUA), scholarships are capped at $5,000 per year. There are approximately 15,000 student-athletes participating in U SPORTS. A recent study found 95% to be enrolled in full-time course load and 83% were undergraduate students, 35% worked outside of school (for an average of 12 h/week and 31% volunteered for an average of 5 h/week; (7)). U SPORTS athletes may also simultaneously participate on provincial, national, and club sport teams. While U SPORTS are a proven pathway to national teams and elite sport in Canada, this may differ by sport.

Over the past decade, there has been an increase in the number of policies and legislation concerning the mental health and wellbeing of student-athletes. For example, in Canada, the university sport governing body endorsed the U SPORT Mental Health Best Practice Guidelines (14). This document outlines for university sport programs what should be provided to support student-athlete mental health, establishing recommendations for (1) ensuring access to qualified and licensed mental health practitioners, (2) ensuring proper procedures to identify student-athletes with mental health concerns and appropriate referral to relevant practitioners, (3) implementing mental health screening into pre-participation examinations, and (4) encouraging sporting environments that support mental wellness and resiliency (14). These guidelines are consistent with those proposed by the NCAA in the United States. The NCAA Mental Health Best Practices document adopts the World Health Organization's definition of mental health as “a state of mental well-being that enables people to cope with the stresses of life, realize their abilities, learn and work well, and contribute to their community” (15). This definition also recognizes mental health exists on a continuum, ranging from mental disorders and illness at one end to mental wellness or flourishing at the other (16, 17).

Policies and services provided for non-athlete students tend to be comparatively less interpersonal and broader - aimed at entire campuses. There is great variation in these services across campuses, as highlighted by the rankings of campus’ mental health services in national publications (e.g., Macleans). These are supplemented by relevant organizations that target the entire national postsecondary population, such as the Centre for Innovation in Campus Mental Health.

While these guidelines represent an important step forward, they are largely deficit-focused, emphasizing reactive strategies to mitigate the consequences of mental ill-being rather than proactive approaches that promote wellbeing. Moreover, these policies are broad and often lack empirically grounded data on the strengths and needs of Canadian student-athletes. To optimize the effectiveness of such policies, there is a need for context-specific research to identify and map the mental health assets of this population.

Deficit-focused, reactive approaches to mental health primarily center on the identification, treatment, and management of mental illness *after* it has manifested. Such approaches are responsive in nature, emphasizing clinical interventions and therapeutic strategies designed to alleviate symptoms and support recovery once the presence of mental illness has been established. The deficit perspective assumes that individuals are lacking mental health, and therefore the goal becomes restoring what is lacking rather than fostering capabilities to maintain mental wellbeing (18). Conversely, proactive approaches to mental health aim to maintain and promote mental wellbeing, prior to the onset of mental illness. These strategies focus on enhancing protective factors that sustain mental wellness, reducing vulnerability to mental health challenges, and mitigating the impact should they arise (19). Within the U SPORTS (14) guidelines, only the strategy of encouraging environments that support mental wellness and resilience qualifies as proactive; the other three recommended strategies adopt a reactive, deficit-oriented orientation. Similarly, the NCAA guidelines (15) outline just one proactive strategy—creating environments that support mental health and promote wellbeing—and explicitly state that athletic programs must maintain a written plan detailing their mental health promotion initiatives.

Asset-based approaches to mental health provide a valuable complement to traditional deficit-focused models. Rather than emphasizing illness and reaction, asset-based frameworks focus on the development and maintenance of individual-level strengths that promote mental health and wellbeing (20–22). Importantly, an asset-based approach aligns closely with the Mental Health Strategy for High Performance Sport in Canada (1), a collaborative policy initiative led by Sport Canada, Canadian Olympic and Paralympic Committees, Own the Podium, elite athletes, and other national stakeholders. This strategy emphasizes not only the treatment of mental illness but also the proactive promotion of mental health through the deliberate cultivation of key assets. By strengthening these capacities, this strategy advances a proactive, evidence-informed model for mental health promotion that seeks to create sustainable environments for athlete flourishing. Although the current U SPORTS (14) and NCAA (15) guidelines do not explicitly reference asset-based principles, their recommendations to create environments that foster wellness suggest support for mental health asset cultivation. Integrating asset-based approaches within existing models may serve student-athletes better by addressing mental illness while simultaneously strengthening the mental health assets needed to maintain long-term wellbeing. Thus, asset-based approaches should be used alongside traditional, deficit-focused models. This underscores the need for research that measures and maps mental health assets within Canadian student-athlete populations.

A wide range of mental health assets may contribute to the wellbeing of student-athletes. The Mental Health Strategy for High Performance Sport in Canada emphasizes mental health literacy, stigma reduction, and help-seeking (1). The U SPORTS guidelines (14) promote mental health literacy, stigma reduction, self-learning, resiliency, coping skills, and positive relationships as important for cultivation in athletic environments. However, it is important to recognize that additional mental health assets may also play a critical role in shaping mental health outcomes in Canadian university sport contexts. In the present study, we focus on social support, resiliency, and mental health resource literacy – three interrelated yet distinct mental health assets that can help student-athletes navigate the challenges of sport and postsecondary life whilst maintaining mental wellbeing.

Social support encompasses the availability and quality of relationships within an individual's social network. Strong social support networks are consistently associated with improved psychological outcomes and reduced risk of mental health difficulties across both general student populations and athletes (23, 24). Within sport contexts, team culture, peer relationships, and coach-athlete interactions represent particularly influential sources of support. Yet, experiences of stigma, pressure to perform, and power dynamics may discourage athletes from fully accessing or relying on these networks (7, 8, 25). Gender differences further shape the role of social support in mental health. Women with greater perceived social support are more likely to experience reductions in distress (26). Further, resiliency acts as a stronger mediator between social support and stress perception for women than men (27). In contrast, men reported lower levels of social support, though the relationship between perceived social support and stress was almost fully mediated by self-perceived resiliency among men (27). Collectively, this evidence underscores that the perception and function of social support differ across gender, shaping mental health outcomes in complex ways. Understanding how student-athletes perceive and experience social support is therefore essential for informing university and sport programming aimed at fostering healthy interpersonal environments.

Resiliency is broadly defined as the ability to withstand and adapt in the face of adversity (28). For student-athletes, resiliency is essential given the cumulative pressures of high-performance environments, including academic workload, athletic demands, injury risk, and the time constraints inherent to university sport participation (7, 29). Assessing resiliency provides insight into individual coping mechanisms and the capacity to sustain positive mental health. Research demonstrates that higher resiliency is associated with greater psychological well-being, adaptive coping, and reduced psychological distress in athletes (30–32). Notably, resiliency appears to function differently across genders: men tend to exhibit higher resiliency levels that correspond with lower perceived stress, whereas women may derive greater stress-buffering benefits from social support than from resiliency alone (27). Understanding resiliency among Canadian student-athletes is therefore essential to cultivate this asset, enhancing adaptive functioning and mental well-being across both academic and athletic domains.

Mental health literacy broadly encompasses the ability to recognize, understand, and manage mental health issues (33). This includes different domains, typically knowledge, attitudes, and resource awareness. Among Canadian student-athletes, research indicates that individuals are relatively aware of available services, yet persistently low rates of service utilization suggest that awareness does not necessarily translate into help-seeking behavior (7, 8, 34). Men are less likely than women to seek mental health support, even when aware of available services, often due to stigma and social norms, and report more comfort in accessing informal supports (e.g., self-help sources from peers) than mental health professionals (23, 35–37). This pattern showcases gender-specific differences in mental health literacy that should be considered. These findings highlight that gendered norms play a role in shaping how mental health knowledge is interpreted and acted upon, particularly in decisions to seek formal support.

The present study sought to bridge the gap between high-level mental health policies and the empirical evidence required for their effective implementation by examining the mental health assets of social support, resiliency, and mental health resource literacy among Canadian university student-athletes and non-athletes, while also examining potential differences by gender. By providing timely evidence of the prevalence of mental health assets within the U SPORTS population, this study aims to inform actionable strategies that enhance student-athlete wellbeing, thereby complementing national mental health policies with asset-based approaches.

This study comprised an analysis of an existing database – the Canadian Campus Wellbeing Survey (CCWS; see description in methods). Because of this, our research question was focused on the mental health assets included in that survey, specifically social support, resiliency, and literacy of mental health resources. Given this is an exploratory study and no research on mental health assets in this population has yet been conducted, no hypotheses were proposed. The purpose of the study was to examine differences in social support, resiliency, and mental health resource literacy between student-athletes and non-athletes. Gender has been noted as an influential factor in the mental health of this population and thus was also included as a factor in these analyses.

The CCWS is a comprehensive, voluntary, and confidential survey of Canadian post-secondary students that covers nine sections related to student health and wellbeing including mental health assets, mental health deficits, and health service utilization/help-seeking. Over 115 Canadian post-secondary institutions have deployed the CCWS. This program provides a necessary basis for assessing the mental health and wellbeing of students.

## Methods

### Design

This research study employed a cross-sectional, quantitative survey design to examine mental health assets in university athletes and non-athletes. Data for this study was drawn from the 2023 deployment of the CCWS, a national survey administered to post-secondary institutions to assess campus climate and student wellbeing. All data was collected using a self-administered online survey. This approach was selected due to the ease of acquiring data from individuals across Canada and at various institutions. The CCWS includes validated measures and custom-designed survey questions.

### Participants

The sample included 1,143 varsity athletes and 24,365 non-athletes. Table 1 summarizes the differences in demographic factors between the two groups.

**Table 1:** Comparison of demographics between varsity athletes and non-athletes.

Demographics	Varsity athlete	Non-athlete
% women*	60.1%	72.6%
% white*	66.1%	52%
% heterosexual*	82.6%	74.1%
% living on campus*	27.0%	13.3%

*p < .05.

### Measures

#### Varsity status and gender

Both varsity status and gender were determined from CCWS demographic questions. Participants were asked if during the past 12 months, they participated in varsity sport, club, or community sport, intramurals, or did not participate in organized sports. For the current analysis, those who self-identified as participating in varsity sport in the past 12 months were considered varsity athletes, and those who reported not having participated in sport in the past 12 months were considered non-athletes. Individuals who did not participate in varsity sport but did participate in club/community or intramural sports were not included in the analyses. This is consistent with previous research on mental health in student-athletes (38).

Gender on the CCWS was measured with the question “What is your gender identity?” Answers included woman, man, non-binary person, Two-Spirit, and I prefer not to answer. Due to the low counts of non-binary, Two-Spirited, and prefer not to answer, particularly within the varsity group, only individuals who self-identified as women or men were included in the analyses.

#### Social support

Social support was measured via the 5-item Social Provision Scale (SPS-5; (39)). The SPS-5 includes five items: (1) I have close relationships that provide me with a sense of emotional security and wellbeing, (2) There is someone I could talk to about important decisions in my life, (3) I have relationships where my competence and skill are recognized, (4) I feel part of a group of people who share my attitudes and beliefs, and (5) There are people I can count on in an emergency. Responses were rated on a 4-point scale from 1 = Strongly disagree to 4 = Strongly agree. Items’ scores were summed, with higher scores reflecting greater perceived social support. Orpana et al. (39) provide support for the scale's validity and reliability. The Cronbach's alpha for the 5-item scale with the current sample was 0.92.

#### Resiliency

Resiliency for this study was defined as an individual's perceived ability to cope with challenges and daily demands. This was a custom-made measure of the CCWS. It was assessed using two items: (1) In general, how would you rate your ability to handle unexpected and difficult problems (a family or personal crisis), and (2) In general, how would you rate your ability to handle day-to-day demands in your life (work, family responsibilities)? Responses were rated on a 5-point Likert scale ranging from 1= Poor to 5 = Excellent. Scores on the two items were summed, with higher scores indicating greater perceived resiliency. The Cronbach's alpha for this 2-item scale with the current sample was 0.77.

#### Mental health resource literacy

The CCWS includes three items that assess the individual's awareness/literacy, specifically regarding perceptions of availability and comfort with mental health resources. This measurement corresponds to resource-oriented literacy, which is a common component of mental health literacy. For example, both the Multicomponent Mental Health Literacy Measure (MMHLM; (40)) and the revised MMHLM (41) include resource-oriented literacy as one of its three primary components. The revised MMHLM (4) and just its resource-oriented factor (42) have both been used with athlete samples.

Specifically with respect to the CCWS, mental health resource literacy was assessed using three items: (1) If I needed to seek professional help for my mental or emotional health, I would know where to access campus-based resources (online or in-person), (2) If I needed to seek professional help for my mental or emotional health, I would know where to go off campus, and (3) How comfortable would you feel talking about personal problems with a professional? Items 1 and 2 were rated on a 6-point Likert scale ranging from 1 = Strongly disagree to 6 = Strongly agree, while item 3 was rated on a 4-point scale ranging from 1 = Not comfortable at all to 4 = Very comfortable. Responses were summed to create a total mental health resource literacy score, with a higher score reflecting greater knowledge and comfort in accessing professional resources. The Cronbach's alpha for this 3-item scale with the current sample was 0.69.

Normative standards from previous CCWS deployment include 52% scoring high (above 15) on Social support, and 70%–77% answering good, very good, or excellent on the Resiliency items (43). There is no normative data on the specific mental health resource literacy items used in the current study.

### Statistical analysis

To answer the exploratory research question, three separate factorial ANOVAs were conducted, one for each mental health asset (i.e., social support, resiliency, and mental health resource literacy). In each, varsity status (athlete or non-athlete) and gender (man or woman) were the independent variables.

All statistical analyses were conducted with IBM SPSS Statistics software (Version 30.0). Correlations among mental health resource literacy, resiliency, and social support ranged from .18–24. While they were significant, they did not reach the threshold of moderate correlations among dependent variables that would justify a MANOVA as opposed to multiple ANOVAS (44). Therefore, analyses examined each variable separately in three independent ANOVAs. Prior to analysis, the assumptions for ANOVA were assessed. Normality of the data was examined using Q-Q plots, and homogeneity of variance was evaluated with Levene's test. Descriptive statistics were calculated for all study variables (see Table 2). Within each ANOVA, significant interactions were followed up with Bonferroni pairwise comparisons within each gender. When no significant interactions were detected, Bonferroni tests were used for *post-hoc* comparisons of significant main effects. Statistical significance was determined at an alpha level of *p* < .05.

**Table 2:** Means and standard deviations for social support, resiliency, and mental health resource literacy by group.

Variable	Men athletes	Men non-athletes	Women athletes	Women non-athletes
Social Support	15.93 (3.57)	15.29 (3.55)	16.46 (3.25)	16.15 (3.20)
Resiliency	7.33 (1.99)	6.52 (2.02)	6.61 (1.76)	6.15 (1.88)
Mental Health Literacy	12.89 (3.76)	12.06 (3.53)	12.60 (3.09)	12.41 (3.25)

Social Support scores can range from 5 to 20, Resiliency scores can range from 2 to 10, Mental Health Resource Literacy scores can range from 3 to 18. For all variables higher scores indicate more of the attribute. Values represent the mean (standard deviation).

## Results

**Figure 1:**
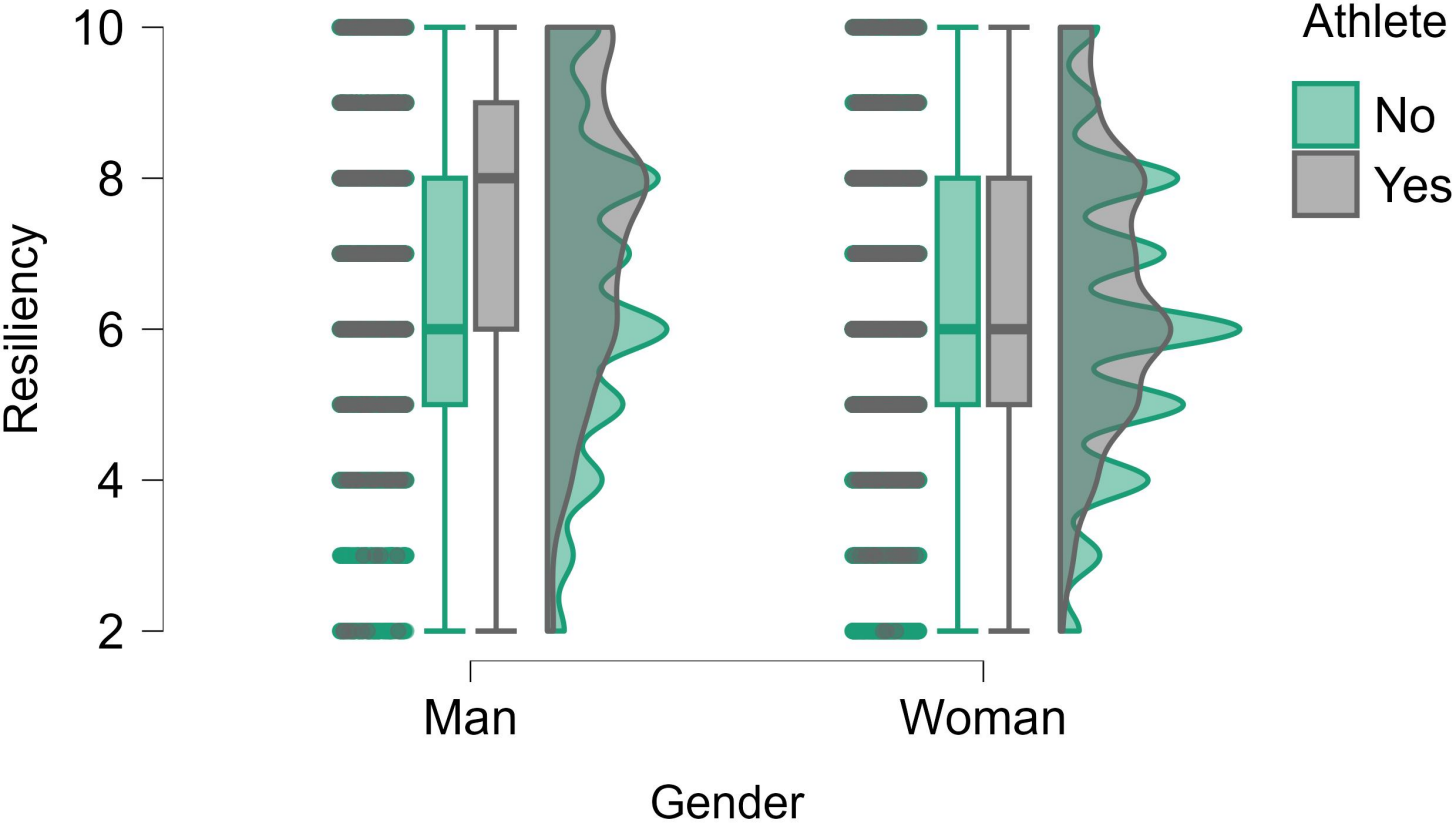
Distribution of resiliency by varsity status and gender.

**Figure 2:**
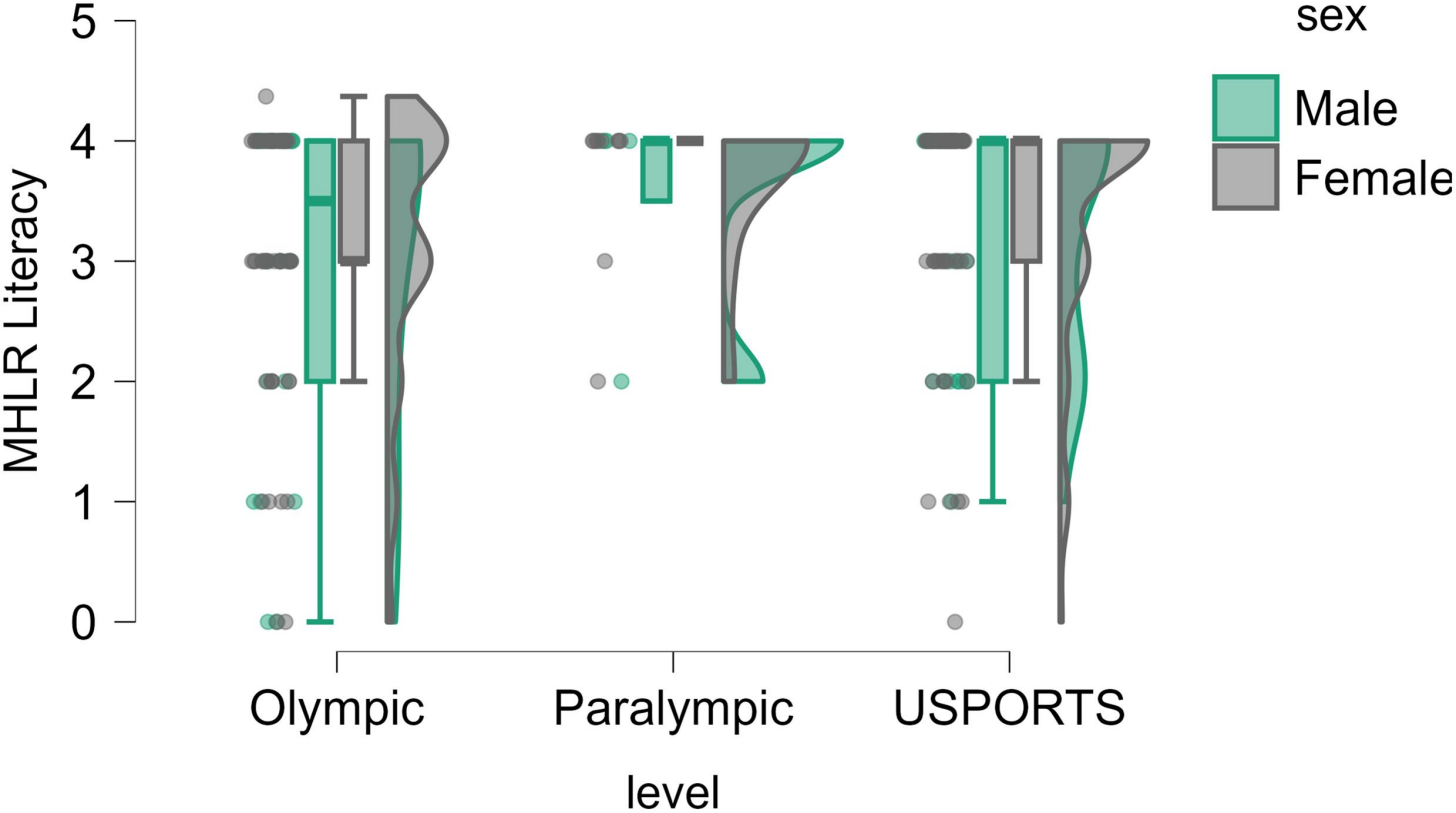
Distribution of mental health resource literacy by varsity status and gender.

**Figure 3:**
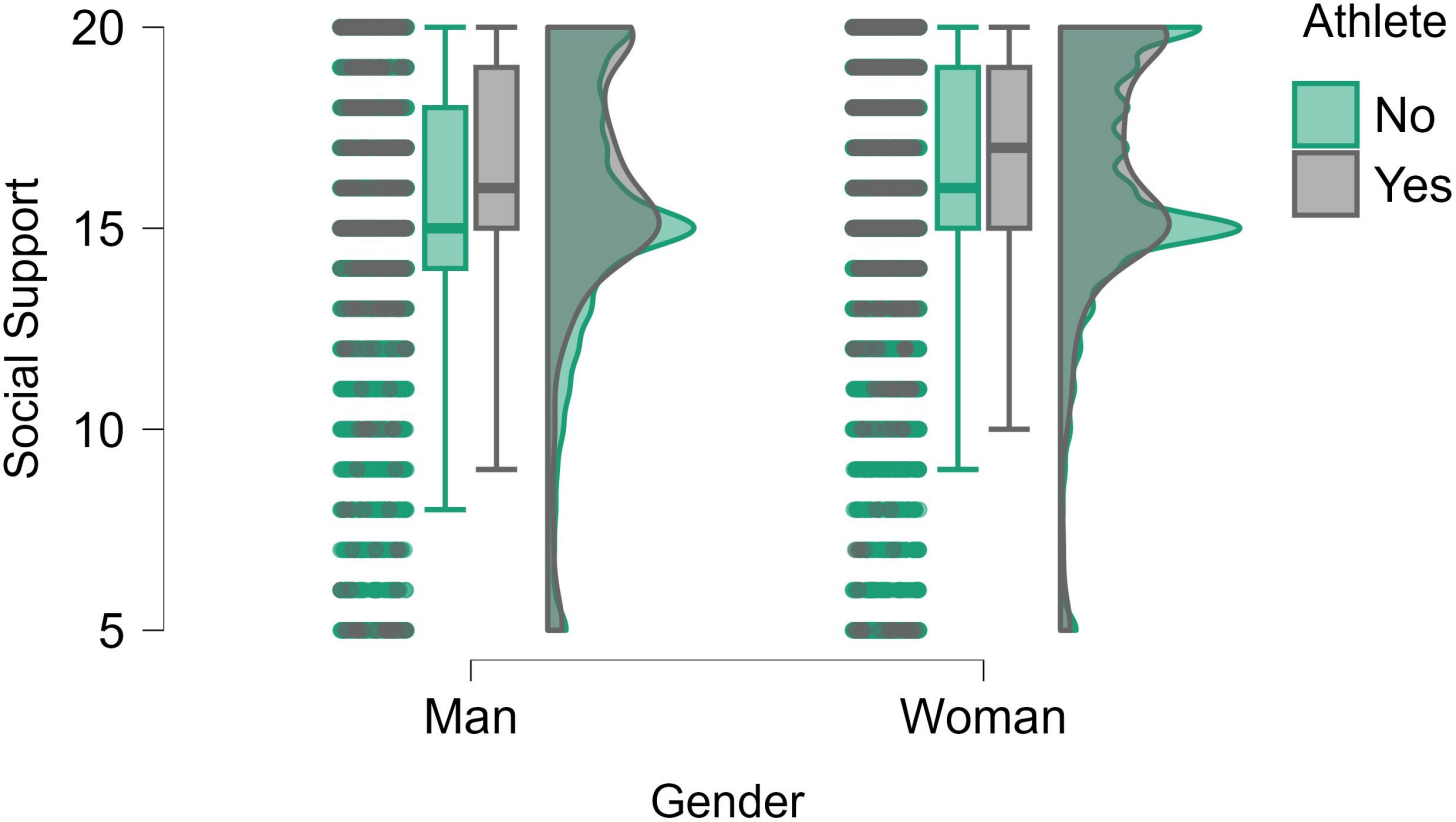
Distribution of social support by varsity status and gender.

Inspection of Q-Q plots for each dependent variable (social support, resiliency, mental health resource literacy) indicated that the data is normally distributed. Homogeneity of variances was assessed with Levene's test, which was significant for all dependent variables – social support: *F*(3, 25,504) = 11.39, *p* < .001; resiliency: *F*(3, 25,504) = 39.21, *p* < .001; and mental health resource literacy: *F*(3, 25,504) = 18.09, *p* < .001. This indicates variance inequality across groups. Given ANOVA's robustness to moderate violations of homogeneity when sample sizes are large (45), the analyses were conducted as planned. Figures 1–3 show the distribution of each measure by gender and athlete status.

### Social support

Results revealed significant main effects of varsity status, *F*(1, 25,504) = 21.64, *p* < .001, *η_p_^2^* = .002, and gender, *F*(1, 25,504) = 45.32, *p* < .001, *η_p_^2^* = .000. The interaction between varsity status and gender was not statistically significant, *F*(1, 25,504) = 32.72, *p* = .099, *η_p_^2^* = .000. *Post hoc* tests showed that women reported significantly more support than men (*t* (1, 25504) = 6.79, *p* < .001, *d* = 0.21), and athletes reported significantly more support than non-athletes (*t* (1, 25504) = 4.65, *p* < .001, *d* = 0.14). Mean scores for all groups were similar to normative values previously reported [i.e., median scores typically greater than 15; (43)].

### Resiliency

There was a significant gender by athlete status interaction for resiliency, *F*(1, 25,504) = 8.51, *p* < .05, *η_p_^2^* = .003. *Post hoc* analyses showed that for both men (*t* (1, 25504) = 8.73, *p* < .001, *d* = 0.42) and women (*t* (1, 25504) = 6.21, *p* < .001, *d* = 0.24), athletes showed significantly higher resiliency, and this trend was significantly greater for men than women. Mean resiliency scores for all groups were also similar to previously reported normative scores, with average scores ranging between good and very good (43).

### Mental health resource literacy

There was a significant gender by athlete status interaction for mental health resource literacy, *F*(1, 25,504) = 9.73, *p* < .05, *η_p_^2^* = .000. *Post hoc* analyses showed among men, there was a statistically significant difference between athletes and non-athletes (*t* (1, 25504) = 5.13, *p* < .001, *d* = 0.25) while there was no statistically significant difference between women athletes and non-athletes.

## Discussion

The present study examined the mental health assets of social support, resiliency, and mental health resource literacy among varsity athletes and non-athletes, with consideration of gender differences. Overall, varsity athletes reported higher levels of mental health assets than their non-athlete peers, although effect sizes were small, and patterns varied by gender. Women reported greater social support than men, and athletes reported greater social support than non-athletes. Men athletes reported the highest levels of resiliency, with athletes generally reporting greater resiliency than non-athletes. Finally, no differences were observed between women athletes and non-athletes in mental health resource literacy, whereas men athletes reported significantly greater resource literacy than men non-athletes. Taken together, these findings contribute to a growing body of evidence suggesting that student-athletes possess meaningful mental health assets, complementing deficit-focused understandings of mental health in postsecondary sport contexts.

Consistent with prior research indicating that sport participation can foster strong social networks, athletes in the present sample reported greater social support than non-athletes (4, 23, 24). While these conclusions should be tempered when considering the effect sizes, participation in organized sport provides structured opportunities for social interaction, including regular team activities, shared goals, and collective identity, which may explain why athletes reported greater support relative to non-athletes. Social support has been identified as a protective factor for mental wellbeing across both general students and athlete populations (4, 23, 24), and particularly influential for women (26). The gender differences in the current study aligns with existing literature suggesting that women are more likely to cultivate, rely on, and seek interpersonal sources of support when managing stress (27, 46). Although men reported lower overall levels of social support, men athletes still reported greater support than non-athletes, further underscoring the role of sport as a socially integrative environment.

The interaction between gender and athlete status on resiliency suggests a more complex pattern. There is conflicting evidence on differences in resiliency between athletes and non-athletes, with other research demonstrating no differences across varsity status (47). However, the current study supports the literature that men report higher resiliency than women (27, 31). Participation in competitive sport environments often entails repeated exposure to performance pressure, injury, evaluation, and adversity, experiences that may foster the development of adaptive coping skills and resiliency over time (29). These demands may partially explain why athletes reported greater resiliency than non-athletes in the present sample.

These findings should be interpreted with caution. Self-reported resiliency may be shaped by gender norms and social desirability, particularly within sport contexts that idealize masculinity, toughness, and emotional control (37, 46, 48). Men athletes may therefore be more likely to endorse resiliency-related items in ways that reflect social expectations rather than lived experiences.

Men athletes reported significantly greater mental health resource literacy than non-athlete peers, whilst no differences were observed among women. Interestingly, this contrasts with previous research indicating that women tend to demonstrate greater awareness of mental health and are more likely to use services (23, 35, 37). Men often hold more negative attitudes toward help-seeking, in part due to gender and societal norms (37, 46, 48). The present results suggest that men athletes are as aware of mental health resources as their women counterparts and potentially comfortable accessing them, whereas men non-athletes are not aware and not comfortable with engaging. Higher resource literacy among athletes may reflect the increased visibility of mental health education and services within athletic programs, as mental health promotion is a central component of mental health sport policy and practice. Both the NCAA and U SPORTS have emphasized mental health education, awareness, and help-seeking as core components of athlete wellbeing initiatives (14, 15). Despite the relatively high resource literacy levels seen in the current results, initiatives to further nurture mental health literacy in this population should still be emphasized. This is important as there are components of mental health literacy that were not assessed by the CCWS (i.e., beliefs and knowledge literacy). Furthermore, mental health literacy as a construct is known to significantly impact stigma and help-seeking (10, 33), which remain high priorities among student-athletes. A resource-oriented approach to mental health literacy remains practically meaningful in the context of policy research.

The lack of observed differences among women may reflect a relatively high baseline of mental health resource literacy within this gender, and within the university population, potentially due to institutional efforts to promote counseling services and campus-wide mental health awareness. It may also indicate that educational strategies are reaching women more effectively, regardless of athlete status. The lower literacy among men non-athletes underscores the need for targeted outreach, particularly given well-documented gender disparities in help-seeking behaviors (23, 36, 37).

The present findings may contribute to a broader shift in student-athlete mental health research toward asset-based models that can complement traditional deficit-focused approaches. This study aligns with evidence suggesting that athletes possess strong protective resources that their non-athlete peers do not hold in the same capacity (4, 23, 24). The current study suggests that student-athletes may have developed stronger social support networks, coping skills, and are more aware of the services available to them. Gender differences observed across assets further underscore the importance of considering how mental health assets are developed, expressed, and reported differently across populations. Clear differences emerged between genders in mental health assets that should not be overlooked.

The primary rationale for this study was to help bridge the gap between policy and practice in intercollegiate sport mental health support. The current results make a significant contribution to the larger picture of student athletes’ mental health. Although the results are somewhat inconsistent, the general pattern regarding mental health deficits/mental ill-health is that student-athletes are as healthy, if not more so, than their non-athlete peers. Policy frameworks within and beyond intercollegiate sport, including those advanced by U SPORTS, the NCAA, and high-performance sport organizations, have increasingly emphasized the importance of fostering environments that promote mental wellbeing, resiliency, and help-seeking (1, 14, 15). Explicitly or not, these policies endorse the cultivation of mental health assets. The present findings provide empirical support for these priorities and highlight specific assets that can be leveraged to inform action.

## Implications

Several institutional and policy implications emerge from these conclusions. First, athletic programs should seek to leverage existing social support networks to normalize conversations about mental health and promote adaptive help-seeking. Peer-based initiatives, team-embedded discussions, and leadership-driven modeling may reinforce already strong social structures and amplify their protective effects. Social networks remain a protective factor against mental illness (4, 23, 24, 27), and are in high supply for athletes. Other initiatives could include mentorship programs, within sports and beyond.

Second, gender-responsive considerations may be warranted. Lower reported resiliency and resource literacy among women suggest a need for programming that supports coping with change, such as during transitions into varsity sport and periods of heightened academic and athletic stress (e.g., during exams and playoffs). Partnerships with non-profit organizations invested in women's athletic experiences may be useful in providing programming and resources related to resiliency for women. At the same time, targeted efforts are needed to engage men, especially non-athletes, in mental health education, addressing cultural norms that may discourage help-seeking. Institutions may benefit from strengthening the visibility of existing resources in male-dominated and athletic spaces.

## Limitations and future directions

Several limitations must be acknowledged. All groups displayed mean scores on these mental health assets that were moderately high and comparable to normative values. Correspondingly, the statistically significant results have quite small effect sizes and probably do not represent practically meaningful differences. Notwithstanding the significant results found, it should still be emphasized that this sample shows relatively high levels of mental health assets and there may not be meaningful differences between athletes and non-athletes, or between genders. However, given the priority of these assets in governing bodies’ statements, and their known impact on mental health behavior outcomes (e.g., resource utilization) we would still encourage further research and action on mental health assets with this population. Although levels are moderately high, there is still room for improvement, which could be meaningful for a population that probably has elevated prevalences of mental health issues (13). The implementation of an asset-based approach combined with efforts to reduce mental health deficits would be a thorough approach that is both consistent with U SPORTS policy, and the dual continuum model of mental health (16).

The use of custom items to assess resiliency and mental health resource literacy may limit comparability with studies using validated scales, and findings should be interpreted in light of the specific constructs assessed. Additionally, reliance on self-report measures introduces the potential for social desirability bias, particularly for constructs such as resiliency that may be shaped by gendered and athletic identity norms (37, 46, 48). Although the large sample size enhanced statistical power, it may have contributed to statistically significant findings with small effect sizes, underscoring the importance of cautious interpretation of practical significance. Finally, it is important to note that this study was conducted on a Canadian sample, which may limit the generalizability of findings to other populations.

Future research should prioritize longitudinal designs to examine how mental health assets develop over time and across key periods in the university experience. The current analysis did not include year of study. This is particularly a limitation when considering potential implications as provisions of resources to student-athletes may be optimized by varying across different years of study. Institution-specific studies are also needed to better understand how contextual factors shape asset development and to inform program-specific policy implementation. Finally, expanding the range of mental health assets examined, such as coping skills, autonomy, and additional dimensions of mental health literacy, will further advance the understanding of mental health assets held by student athletes.

## Data Availability

The data analyzed in this study is subject to the following licenses/restrictions: Data was complied by the Canadian Campus Wellbeing Survey. Individual researchers can apply for data access. Requests to access these datasets should be directed to https://www.ccws-becc.ca/data-access-policy. The data used for this research were made available by the Rossy Foundation. We thank the students and institutions that participated in the CCWS.

## References

[1] Durand-Bush N Van Slingerland K. *Mental health strategy for high performance sport in Canada*. The Mental Health Partner Group [Canadian Centre for Mental Health and Sport, Canadian Olympic and Paralympic Sport Institute Network, Game Plan, and Own The Podium] (2021).

[2] Schinke RJ Stambulova NB Si G Moore Z. International Society of Sport Psychology position stand: athletes’ mental health, performance, and development. Int J Sport Exerc Psychol. (2018) 16:622–39. 10.1080/1612197X.2017.1295557

[3] Adlaf EM Gliksman L Demers A Newton-Taylor B. The prevalence of elevated psychological distress among Canadian undergraduates: findings from the 1998 Canadian campus survey. J Am Coll Health. (2001) 50(2):67–72. 10.1080/07448480109596009 11590985

[4] Armstrong S Oomen-Early J. Social connectedness, self-esteem, and depression symptomatology among collegiate athletes versus non-athletes. J Am Coll Health. (2009) 57(5):521–6. 10.3200/JACH.57.5.521-526 19254893

[5] Linden B Boyes R Stuart H. Cross-sectional trend analysis of the NCHA II survey data on Canadian post-secondary student mental health and wellbeing from 2013 to 2019. BMC Public Health. (2021) 21(1):590. 10.1186/s12889-021-10622-1 33765965 PMC7992810

[6] American College Health Association. American College Health Association-National College Health Assessment II: Canadian Consortium Executive Summary Spring 2019. Silver Spring, MD: American College Health Association (2019).

[7] Giovannetti SL Robertson JRG Colquhoun HL Malachowski CK. Mental health services for Canadian university student-athletes: an exploratory survey. J Clin Sport Psychol. (2019) 13(3):469–85. 10.1123/jcsp.2018-0048

[8] Gulliver A Griffiths KM Christensen H. Barriers and facilitators to mental health help-seeking for young elite athletes: a qualitative study. BMC Psychiatry. (2012) 12(1):157. 10.1186/1471-244X-12-157 23009161 PMC3514142

[9] Davoren AK Hwang S. “Depression and anxiety prevalence in student-athletes”. In: Brown GT, editor. Mind, Body and Sport: Understanding and Supporting Student-athlete mental Wellness. Indianapolis: National Collegiate Athletic Association (2014). p. 38–9.

[10] Sullivan P Blacker M Murphy J Cairney J. Levels of psychological distress of Canadian university student-athletes. Can J High Educ. (2019) 49(1):47–59. 10.7202/1060823ar

[11] Harris BR Maher BM. Student-athlete mental health, help-seeking, and service utilization: implications for a multi-tiered, public health approach on college campuses. J College Stud Psychother. (2023) 37(4):371–90. 10.1080/87568225.2022.2109548

[12] Kilcullen JR Scofield BE Cummins AL Carr BM. Collegiate athlete mental health: comparing treatment-seeking student-athletes and nonathletes on service utilization, clinical presentation, and outcomes. Sport Exerc Perform Psychol. (2022) 11(2):138–55. 10.1037/spy0000280

[13] Kegelaers J Wylleman P Defruyt S Praet L Stambulova N Torregrossa M. The mental health of student-athletes: a systematic scoping review. Int Rev Sport Exerc Psychol. (2024) 17(2):848–81. 10.1080/1750984X.2022.2095657

[14] U SPORTS. Mental Health Best Practices (2020).

[15] National Collegiate Athletic Association. Mental Health Best Practices: Understanding and Supporting Student-Athlete Mental Health. NCAA.org (2024).

[16] Keyes CL. The mental health continuum: from languishing to flourishing in life. J Health Soc Behav. (2002) 43:207–22. 10.2307/3090197 12096700

[17] Keyes CL Simoes EJ. To flourish or not: positive mental health and all-cause mortality. Am J Public Health. (2012) 102:2164–72. 10.2105/AJPH.2012.300918 22994191 PMC3477942

[18] Anglin DM Polanco-Roman L. “Deficit model”. In: Zeigler-Hill V Shackelford TK, editors. The SAGE Encyclopedia of Abnormal and Clinical Psychology (Vol. 7). Thousand Oaks, CA: SAGE Publications (2017). p. 975. 10.4135/9781483365817.n381

[19] Singh V Kumar A Gupta S. Mental health prevention and promotion—a narrative review. Front Psychiatry. (2022) 13:898009. 10.3389/fpsyt.2022.898009 35958637 PMC9360426

[20] Lightfoot E McCleary JS Lum T. Asset mapping as a research tool for community-based participatory research in social work. Soc Work Res. (2014) 38:59–64. 10.1093/swr/svu001

[21] Rotegård AK Moore SM Fagermoen MS Ruland CM. Health assets: a concept analysis. Int J Nurs Stud. (2010) 47:513–25. 10.1016/j.ijnurstu.2009.09.005 19819452

[22] Whiting L Kendall S Wills W. An asset-based approach: an alternative health promotion strategy. Community Pract. (2012) 85:25–8.23029774

[23] Campbell F Blank L Cantrell A Baxter S Blackmore C Dixon J. Factors that influence mental health of university and college students in the UK: a systematic review. BMC Public Health. (2022) 22(1):1778. 10.1186/s12889-022-13943-x 36123714 PMC9484851

[24] Luo J Du R Wang X Luo L. The relationship between social support and mental health in athletes: a systematic review and meta-analysis. Front Psychol. (2025) 16:1642886. 10.3389/fpsyg.2025.1642886 40969465 PMC12442422

[25] Rao AL Hong E. “Overcoming the stigma of mental health in sport”. In: Hong E Rao A, editors. Mental Health in the Athlete. Cham: Springer (2020). p. 1–10. 10.1007/978-3-030-44754-0_1

[26] Canadian Institute for Health Information, and Canadian Electronic Library. The role of social support in reducing psychological distress. Canadian Institute for Health Information (2012).

[27] Yalcin-Siedentopf N Pichler T Welte AS Hoertnagl CM Klasen CC Kemmler G. Sex matters: stress perception and the relevance of resilience and perceived social support in emerging adults. Arch Womens Ment Health. (2021) 24(3):403–11. 10.1007/s00737-020-01076-2 33057788 PMC8116239

[28] Gupta S McCarthy PJ. The sporting resilience model: a systematic review of resilience in performers. Front Psychol. (2022) 13:1003053. 10.3389/fpsyg.2022.1003053 36619099 PMC9811683

[29] Sarkar M Fletcher D. Psychological resilience in sport performers: a review of stressors and protective factors. J Sports Sci. (2014) 32(15):1419–34. 10.1080/02640414.2014.901551 24716648

[30] Chuang SP Wu JYW Wang CS. Resilience and quality of life in people with mental illness: a systematic review and meta-analysis. Neuropsychiatr Dis Treat. (2023) 19:507–14. 10.2147/NDT.S392332 36910331 PMC9994666

[31] Konaszewski K Niesiobędzka M Surzykiewicz J. Resilience and mental health among juveniles: role of strategies for coping with stress. Health Qual Life Outcomes. (2021) 19(1):58. 10.1186/s12955-021-01701-3 33602278 PMC7891003

[32] Hosseini SA Besharat MA. Relation of resilience with sport achievement and mental health in a sample of athletes. Procedia Soc Behav Sci. (2010) 5:633–8. 10.1016/j.sbspro.2010.07.156

[33] Jorm AF Korten AE Jacomb PA Christensen H Rodgers B Pollitt P. “Mental health literacy”: a survey of the public’s ability to recognise mental disorders and their beliefs about the effectiveness of treatment. Med J Aust. (1997) 166:182–6. 10.5694/j.1326-5377.1997.tb140071.x 9066546

[34] López RL Levy JJ. Student athletes’ perceived barriers to and preferences for seeking counseling. J Coll Couns. (2013) 16(1):19–31. 10.1002/j.2161-1882.2013.00024.x

[35] Rafal G Gatto A DeBate R. Mental health literacy, stigma, and help-seeking behaviors among male college students. J Am Coll Health. (2018) 66(4):284–91. 10.1080/07448481.2018.1434780 29419361

[36] Thompson AE Anisimowicz Y Miedema B Hogg W Wodchis WP Aubrey-Bassle K. The influence of gender and other patient characteristics on health care-seeking behaviour: a QUALICOPC study. BMC Fam Pract. (2016) 17:38. 10.1186/s12875-016-0440-0 27036116 PMC4815064

[37] Wong K. Gender differences in mental health literacy of university students. West Undergrad Psychol J. (2016) 4(1):1–11.

[38] Anchuri K Davoren AK Shanahan A Torres M Wilcox HC. Nonsuicidal self-injury, suicidal ideation, and suicide attempt among collegiate athletes: findings from the National College Health Assessment. J Am Coll Health. (2020) 68(8):815–23. 10.1080/07448481.2019.1616743 31210585

[39] Orpana HM Lang JJ Yurkowski K. Validation of a brief version of the Social Provisions Scale using Canadian national survey data. Health Promot Chronic Dis Prev Can. (2019) 39(12):323–32. 10.24095/hpcdp.39.12.02 31825785 PMC6938275

[40] Jung H Von Sternberg K Davis K. Expanding a measure of mental health literacy: development and validation of a multicomponent mental health literacy measure. Psychiatry Res. (2016) 243:278–86. 10.1016/j.psychres.2016.06.034 27423635

[41] Sullivan P Murphy J Blacker M. The psychometric properties of the multicomponent mental health literacy measure with a sample of student-athletes and student athletic therapists. J Appl Sport Psychol. (2021) 33:343–56. 10.1080/10413200.2019.1702119

[42] O'Connor J Jeanes R Lambert K Bevan N Young L Powers T. The impact of a mental health literacy program on sporting club environment, member confidence and knowledge to support. Ment Health Prev. (2024) 33:200326. 10.1016/j.mhp.2024.200326

[43] Derkach L Wu C Faulkner G (2025). National Snapshot: Fall 2024 - Winter 2025. Student version (CCWS Technical Report Series 2025; No. 2). Canadian Campus Wellbeing Survey, The University of British Columbia. Available online at: https://www.ccws-becc.ca (Accessed March 4, 2025).

[44] Field A. Discovering Statistics Using IBM SPSS Statistics. 6th ed London: SAGE Publications (2024).

[45] Tabachnick BG Fidell LS. Using Multivariate Statistics. 7th ed Boston, MA: Pearson (2018).

[46] Khan A Zeb I Fang S. Gender differences in mental health experiences and perceived social support among university students: a qualitative case study. Int J Qual Stud Health Well-being. (2025) 20(1):2576004. 10.1080/17482631.2025.2576004 41176709 PMC12581782

[47] Knowles C Shannon S Prentice G Breslin G. Comparing mental health of athletes and non-athletes as they emerge from a COVID-19 pandemic lockdown. Front Sports Act Living. (2021) 3:612532. 10.3389/fspor.2021.612532 34095823 PMC8172612

[48] Steinfeldt JA Steinfeldt MC. Profile of masculine norms and help-seeking stigma in college football. Sport Exerc Perform Psychol. (2012) 1(1):58–71. 10.1037/a0024919

